# Effects of lactoferrin on neonatal pathogens and *Bifidobacterium breve* in human breast milk

**DOI:** 10.1371/journal.pone.0201819

**Published:** 2018-08-22

**Authors:** Tabitha Woodman, Tobias Strunk, Sanjay Patole, Benjamin Hartmann, Karen Simmer, Andrew Currie

**Affiliations:** 1 Medical & Molecular Sciences, Murdoch University, Perth, Western Australia; 2 Centre for Neonatal Research and Education, School of Medicine, University of Western Australia, Perth, Western Australia; 3 Neonatal Directorate, King Edward Memorial Hospital for Women, Perth, Western Australia; 4 The Perron Rotary Express Milk Bank, King Edward Memorial Hospital for Women, Perth, Western Australia; Centre Hospitalier Universitaire Vaudois, FRANCE

## Abstract

Supplementation with probiotics in preterm infants reduces necrotizing enterocolitis and sepsis. Bovine lactoferrin is a promising supplement that may further reduce disease burden, but its effects on probiotic bacteria in human breast milk has not been evaluated. We aimed to characterise the antimicrobial activity of bovine and human lactoferrin in human breast milk against probiotics and typical neonatal sepsis pathogens. Lactoferrin levels were determined by enzyme linked immunosorbent assay in fresh and pasteurised human breast milk. The neonatal pathogens *Staphylococcus epidermidis* and *Escherichia coli*, and the probiotic *Bifidobacterium breve* strain M-16V were cultured in human breast milk or infant formula in the presence or absence of clinically relevant doses of bovine or human lactoferrin. Standard microbiological methods were used to determine the effects of lactoferrin on bacterial growth. Unpasteurised human breast milk contained significantly higher lactoferrin levels and resulted in superior inhibition of pathogenic bacterial growth compared to infant formula and pasteurised human breast milk. Human lactoferrin was significantly more effective at inhibiting bacterial growth, when compared to bovine lactoferrin. Supplementation with human lactoferrin or high dose bovine lactoferrin inhibited growth of the probiotic strain *B*. *breve* M-16V in pasteurised human breast milk. Although unpasteurised human breast milk and human lactoferrin had the greatest antimicrobial activity against all bacterial species tested, higher doses of bovine lactoferrin also showed activity against *B*. *breve* and. *S*. *epidermidis*. This study suggests that simultaneous administration of lactoferrins and probiotics may affect colonisation with probiotic bacteria, warranting further investigations.

## Introduction

Preterm neonates are highly susceptible to infectious and inflammatory diseases such as late-onset sepsis (LOS) and necrotising enterocolitis (NEC) and suffer high morbidity, including long term sequelae [1]. Dysbiosis of the preterm infant gastrointestinal tract is a major factor contributing to the development of LOS and NEC [2–5]. Gut dysbiosis, a loss of microbial diversity and overgrowth of Proteobacteria in the small intestine [6, 7], can lead to a cascade of inflammation and translocation of pathogens into the bloodstream [4].

Prevention of gut dysbiosis therefore is critical to preventing infectious and inflammatory diseases such as LOS and NEC. Human breast milk feeding is beneficial in preterm neonates and promotes development of a healthy intestinal microbiome, along with passive immune protection via a variety of soluble and cellular components [8, 9]. Lactoferrin (Lf) is a major antimicrobial protein found naturally in human breast milk. The antimicrobial activity of Lf comes from its ability to directly lyse microbes and to sequester iron and make it unavailable for microbial growth [10, 11]. Lf also has anti-inflammatory and immunomodulatory effects within the gut [12]. Due to these properties, Lf may significantly decrease the burden of both LOS and NEC, when supplemented to preterm neonates by controlling the overgrowth of sepsis causing pathogens and decreasing inflammation in the gut [13].

A higher consumption volume of breast milk in preterm neonates correlates with a lowered risk of LOS and NEC [9]. Ongoing clinical trials, such as the Lactoferrin Infant Feeding Trial (LIFT; ACTRN12611000247976) and Enteral Lactoferrin in Neonates (ELFIN; ISRCTN88261002 [13]), are evaluating the benefits of high dose supplementation of breast milk with bovine Lf (bLf) to prevent and limit the impact of LOS and NEC. Probiotics are beneficial live microbes which when administered can promote microbiota diversity, improve gut barrier function and colonisation with other healthy commensals [14]. When administered to preterm neonates, probiotics can seed the lower gut and decrease the burden of LOS and NEC by decreasing the risk of inflammation, dysbiosis and translocation of pathogens [15–17].

Colonisation with probiotic bacteria, and simultaneous control of the overgrowth of sepsis pathogens in the preterm gut, may be critical for preventing LOS and for dampening inflammation. Supplementation of enteral feeds with probiotics or bLf has shown positive clinical outcomes for LOS and NEC prevention. One clinical trial has combined these two interventions and shown a 4.6% decrease in LOS cases. There were also no cases of NEC in this study in the combination treatment group, compared to the 6% incidence in the placebo group [16]. Despite these positive clinical outcomes, this trial did not examine whether there was an additive benefit by co-administration of these two supplements.

With supplementation of probiotics now routine in many neonatal intensive care units (NICU) in high-resource settings [18–20] and bLf supplementation under clinical evaluation, there is an urgent need to characterise potential interactions between these two interventions. To our knowledge there have been no *in vitro* studies which have examined whether probiotics and Lf are compatible to be administered together as supplements to milk feeds. This is especially relevant when considering the inherent antimicrobial activity of breast milk [9]. Boosting Lf levels in human breast milk could negatively affect the viability of gut seeding probiotics. Conversely, the presence of probiotic bacteria could limit the activity of Lf against LOS and NEC pathogens in milk. Here, we examined the growth of the probiotic strain *Bifidobacterium breve* M-16V (the routine probiotic used in our NICU) and the common neonatal pathogens *Staphylococcus epidermidis* and *Escherichia coli* in pasteurised and unpasteurised human breast milk and low birth weight infant formula. Pasteurised human breast milk was included in this study as this is commonly fed to preterm infants in the NICU when their mother’s own milk is not available or in inadequate supply. Pasteurisation of donated human breast milk is common practice to eliminate potential sources of pathogens to the preterm infant [21]. We then characterised how bacterial growth is affected by supplementation of pasteurised and unpasteurised human breast milk with bovine and human Lf (bLf and hLf).

## Methods

### Milk collection and processing

Human breast milk samples were donated by five healthy mothers as part of routine milk banking with the Perron Rotary Express Milk (PREM) Bank, King Edward Memorial Hospital, Perth, Western Australia [21]. Before donating expressed breast milk, donors must answer questions relating to the use of prescription medication, smoking and alcohol consumption. Donors are also screened for the infectious diseases HIV, Hepatitis C and B, Human T cell Lymphotrophic virus I and II and Syphilis and are screened every 3 months. Collection of samples was approved by the Women and Newborn Health Services Ethics Committee & Research Governance Office (2015092EW) and all procedures were performed in adherence with best practice guidelines established for human milk banks in Australia [21]. Informed consent was obtained from all mothers for the use of their milk sample for research purposes, prior to milk donation and banking. Donated human breast milk samples were supplied as anonymized samples by the PREM Bank for this study, and expressed into a sterile container and split into two pools for each donor to be pasteurised (PDHM) or left unpasteurised (UDHM). Samples that were pasteurised were exposed to temperatures of 62.5°C in a water bath for 30 minutes, as per the Holder pasteurisation technique routinely used in milk banks [21]. The PREM Bank examines donated human breast milk for microbes both before and after the pasteurisation process. To be accepted into the PREM bank, pasteurised donated human breast milk must not show any level of microbial growth, whereas unpasteurised donated human breast milk must not exceed 1x10^5^ colony forming units per mL (CFU/mL), must not contain Enterobacteriaceae, enterococci or any pathogens capable of producing heat stable enterotoxins.

Pre-NAN Gold preterm and low birth weight infant formula (Nestlé, Vevey, Switzerland) was included as a growth medium for positive bacterial growth, as this formula mimics the complex mixture of sugars, lipids and proteins found in human milk, but contains no antimicrobial proteins or peptides that may inhibit bacterial growth. Infant formula and donated human breast milk samples were aliquoted into 5mL sterile tubes and stored as non-defatted milk in a monitored -20°C freezer until required for experimentation.

### Quantification of lactoferrin levels in human breast milk samples

The concentration of Lf in the PDHM and UDHM was quantified using a sandwich ELISA method [9]. The antibodies used for the capture and detection of human Lf were mouse IgG_1_ anti-human Lf (Abcam, Cambridge, United Kingdom, clone 2B8; ab10110) and biotinylated rabbit polyclonal IgG anti-human Lf (Abcam, ab25811), both with a final concentration of 0.5μg/mL. A standard curve was prepared using serial 2-fold dilutions of purified hLf (Aviva systems biology, San Diego, California, United States) and used to interpolate the Lf concentrations in milk samples using a five parameter logistic curve fit. All milk samples were diluted to 1 in 2x10^6^ for this assay. Avidin-HRP (eBioscience, San Diego, California, United States) was added to the biotinylated antibodies for detection, and TMB substrate (eBioscience) for development and 1M orthophosphoric acid (ChemSupply, Gillman, Australia) to stop the reaction. Absorbance of duplicate samples were measured at 450nm on a plate reading spectrophotometer (Multiskan^™^ FC Microplate Photometer, ThermoFisher Scientific, Malaga, Western Australia) and samples below the limit of detection were assigned an arbitrary concentration equal to half of the lowest value of the standard curve, 7.8125x10^-4^ mg/mL.

### Preparation of bacterial stocks

The bacterial strains used in this study were *Staphylococcus epidermidis* WT 1457 (supplied by Dr Michael Otto, National Institutes of Health, United States), *Escherichia coli* ATCC 11775 (American Type Culture Collection, Virginia, United States) and *Bifidobacterium breve* M-16V (Morinaga & Company, Ltd, Tokyo, Japan). Frozen mid-log stocks were prepared for each bacteria according to our previously published method [22]. *S*. *epidermidis* and *E*. *coli* isolates were streaked out onto blood agar (PathWest Laboratory Medicine WA Media; Mt Claremont, Australia) and *B*. *breve* onto reinforced clostridial agar (RCA; Oxoid, Hampshire, England). Individual colonies of *S*. *epidermidis* and *E*. *coli* were then incubated aerobically at 37°C overnight, while *B*. *breve* was incubated anaerobically at 37°C for 48 hours. After incubation, overnight/48 hour cultures were inoculated separately into either 15mL of Heart Infusion broth (Sigma-Aldrich, Castle Hill, Australia) for *S*. *epidermidis*, Luria Bertani broth (Sigma-Aldrich) for *E*. *coli* or into 50mL of fresh de Man, Rogosa and Sharp (MRS; Sigma-Aldrich) broth supplemented with 0.05% (w/w) L-cysteine HCl (Sigma-Aldrich) for *B*. *breve*. The *B*. *breve* starting density was adjusted to an optical density (OD) of 0.15 at 600nm (BioPhotometer^®^ D30, Eppendorf, Hamburg, Germany) and incubated without shaking at 37°C. The starting densities of *S*. *epidermidis* and *E*. *coli* were adjusted to an OD of 0.05 at 600nm and incubated again, as above. Bacterial growth was measured until the OD readings reached the mid-logarithmic point for *S*. *epidermidis* (OD = 0.6 after 155 minutes of incubation), *E*. *coli* (OD = 0.36 after 75 minutes of incubation) and *B*. *breve* (OD = 0.7 after 6 hours of incubation). All aggregated bacteria and debris was removed by centrifugation at 60 x *g* for two minutes from the mid-log cultures. Sterile 80% glycerol (Sigma-Aldrich) was added to the *S*. *epidermidis* and *B*. *breve* suspensions to give a final concentration of 10% (v/v) glycerol. Fetal calf serum (Sigma-Aldrich) was added to *E*. *coli* suspensions to a final concentration of 20% (v/v). Bacteria were stored at -80°C for no more than 3 months prior to use. The viability of mid-log stocks was evaluated after initial freezing and found to be >90% for *S*. *epidermidis* and *B*. *breve and 37% for E*. *coli*. No further loss in viability was seen with the remaining frozen stocks over the course of all experiments. This initital viability measurement was used to adjust the starting inocula for all experiments and verified on the day of inoculation of each milk sample using our previously described colony counting method [9]. Briefly, to determine CFU/mL, each sample was serially diluted from 1x10^-1^ to 1x10^-6^ in sterile phosphate buffered saline (PBS; Life Technologies, Victoria, Australia). Each dilution was then spotted out in 10μL triplicates onto blood agar (for *S*. *epidermidis* and *E*. *coli*) or RCA (for *B*. *breve*) and incubated aerobically overnight (for *S*. *epidermidis* and *E*. *coli*) or anaerobically for 48 hours (for *B*. *breve*) at 37°C. Dilutions of samples which resulted in 10–100 visible colonies where manually counted in order to calculate CFU/mL.

### Growth inhibition assay

The frozen stock of *B*. *breve*, *E*. *coli* and *S*. *epidermidis* were thawed and washed three times by centrifugation at 4000 x *g* in sterile PBS (Life Technologies, Victoria, Australia). The concentration was adjusted so that the final inoculum in each condition was 1x10^6^ CFU/mL. Bovine lactoferrin (bLf, Dicofarm, Rome, Italy) and milk derived human lactoferrin (hLf, Athens Research and Technology, Georgia, United States) was dissolved into sterile PBS (Life Technologies). Reconstituted Lf was then supplemented into both UDHM and PDHM to make the final adjusted concentrations to 5mg/mL and 50mg/mL bLf, and 5mg/mL hLf. The doses of bLf were based on typical concentrations of bLf administered to preterm neonates in current clinical trials (LIFT, ACTRN12611000247976; ELFIN, ISRCTN 88261002). The dose of hLf used was previously reported to be bacteriostatic against a range of sepsis-causing pathogens and reflected the median Lf levels in human breast milk of mothers delivering preterm, one week after delivery [9].

Each experimental condition was inoculated with 1x10^6^ CFU/mL of *B*. *breve*, *S*. *epidermidis* or *E*. *coli*. Each condition was also repeated with co-culture of 1x10^6^ CFU/mL *B*. *breve* and *S*. *epidermidis* or 1x10^6^ CFU/mL *B*. *breve* and *E*. *coli*. Infant formula was used as a growth control for all bacteria for comparison. The milk, Lf and bacteria inoculation was mixed thoroughly in a sterile 96-well polypropylene plate and incubated for four hours at 37°C in a 5% CO_2_ humidified incubator. After four hours of incubation, the culture from each condition, along with the starting inoculum from time zero, was spotted in triplicate onto non-selective blood agar plates or RCA and enumerated by a colony count method, as described above, to determine remaining CFU/mL [9]. We also spotted out untreated milk (UPDHM) samples for enumeration of possible contaminating bacteria on both blood agar (aerobic) and reinforced clostridial agar (anaerobic) and found there were no detectable levels of bacteria present (data not shown), justifying the use of non-selective agar for CFU determinations. Growth inhibition capacity of different milk types was calculated by comparing the remaining live bacteria concentration in each milk type (LBWF, PDHM and UPDHM) and analysed for statistical significance as described below. The effect of different Lf forms on bacterial growth inhibition was calculated by comparing the remaining live bacteria concentration in each milk type spiked with Lf to unspiked milk of the same type, and analysed for statistical significance as described below.

### Statistical analysis

All data were analysed using the statistical data analysis software in GraphPad Prism^®^ v5 (San Diego, California, United States). Non-parametric tests were used due to the small sample size (n = 5). Group comparisons were made using Wilcoxon matched-pairs signed rank test. All results from statistical tests were considered significant if the *p*-value was <0.05.

## Results

### Quantification of lactoferrin in milk

**Fig 1:**
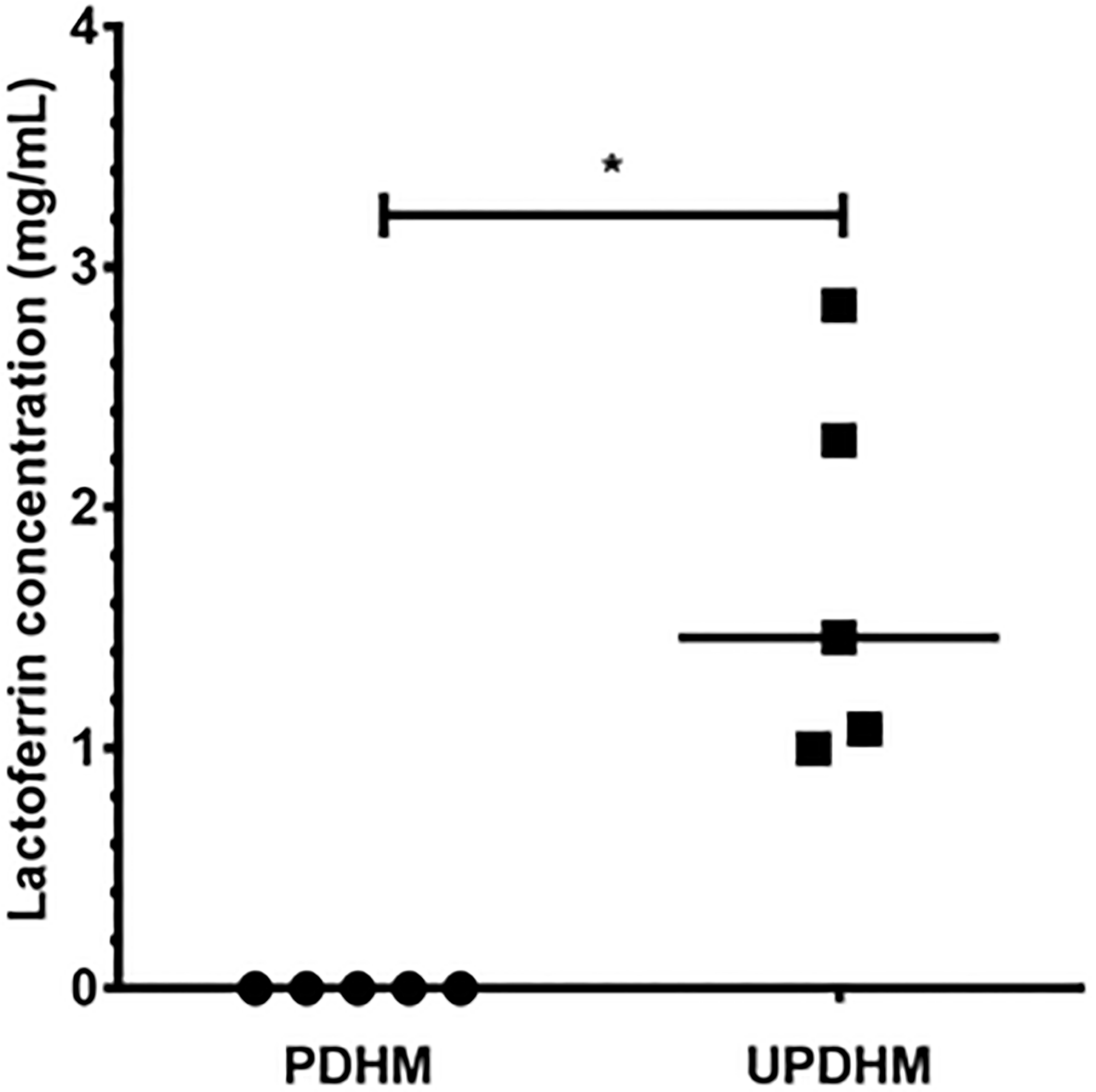
Detectable lactoferrin (Lf) in pasteurised (PDHM) and unpasteurised (UDHM) donated human breast milk samples. Data expressed as mg/mL of Lf. Where there were undetectable quantities of Lf, samples were given an arbitrary value of 7.8125x10^-4^ mg/mL Lf, which was the lowest point detected on the standard curve. Data show median (n = 5), *p <0.05 Wilcoxon matched-pairs signed rank test.

The concentration of Lf found in UDHM ranged from 0.99mg/mL to 2.84mg/mL (median = 1.46mg/mL), whereas there was no detectable Lf in PDHM samples at the dilution tested (*p* = 0.03; Fig 1).

### Growth of bacteria in human breast milk and infant formula

**Fig 2:**
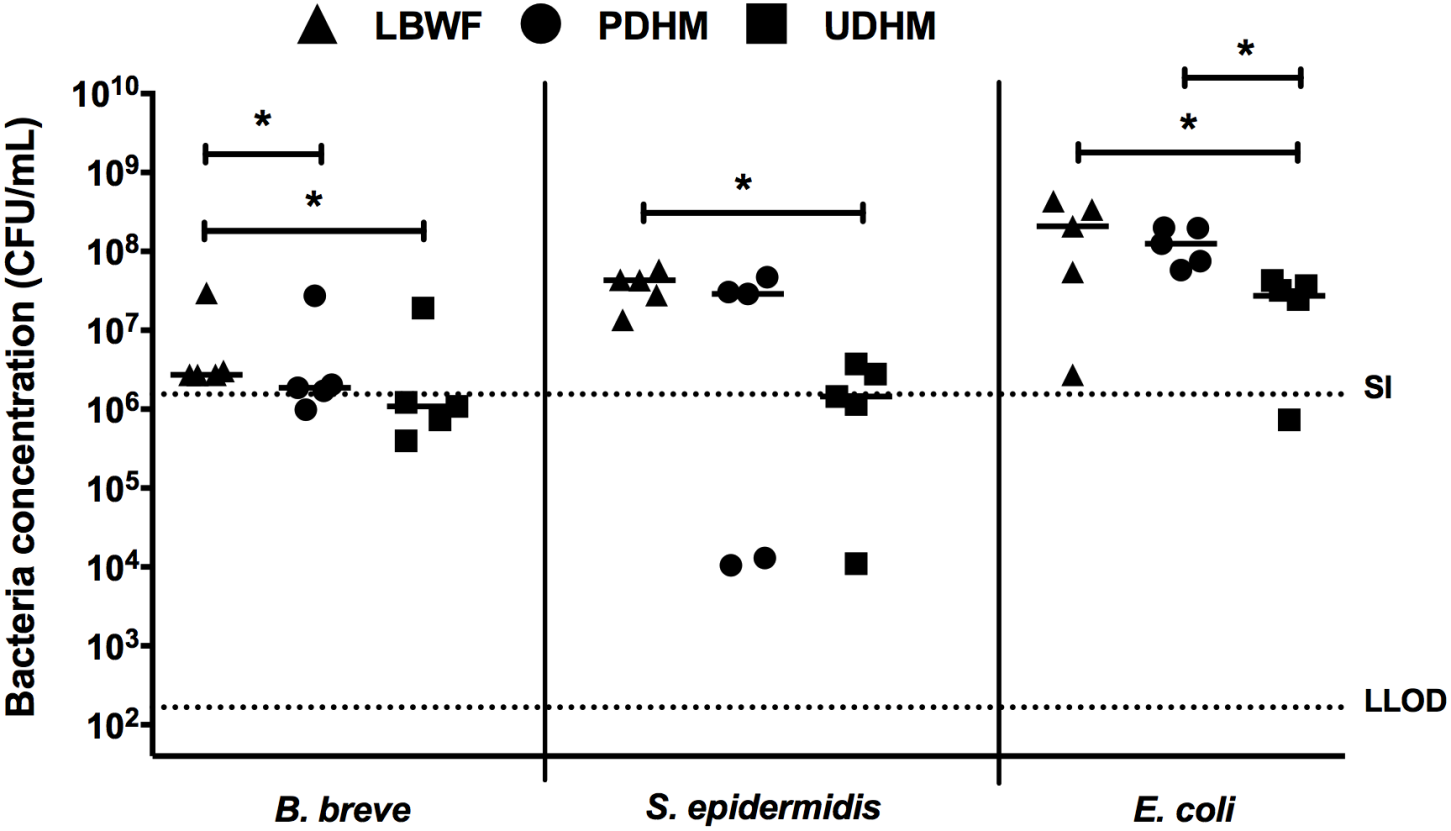
Bacterial growth of *B*. *breve*, *S*. *epidermidis* and *E*. *coli* in Low Birth Weight Formula (LBWF), pasteurised and unpasteurised donated human breast milk (PDHM and UDHM). Results from five experiments showing median of the remaining CFU/mL after 4 hours incubation in each milk. The starting inoculum was quantified at the start of each experiment and the median starting inoculum (SI) and lower limit of detection (LLOD) over the five experiments are indicated by the dotten lines. *p<0.05; comparing remaining bacterial concentration (CFU/mL) in each milk by Wilcoxon matched-pairs signed rank test.

We then examined the growth of *B*. *breve*, *S*. *epidermidis* and *E*. *coli* in PDHM, UDHM and infant formula. In infant formula, *B*. *breve* showed 0.2 log-fold growth after 4 hours, but growth was inhibited in PDHM and was only able to grow 0.1 log-fold after 4 hours (*p* = 0.03; Fig 2). This effect was more pronounced in UDHM, and *B*. *breve* did not show any growth above the starting inoculum (*p* = 0.03; Fig 2). *S*. *epidermidis* grew 1.5 log-fold in infant formula after 4 hours. *S*. *epidermidis* growth was unaffected by PDHM, whereas it was significantly inhibited by UDHM, with the median CFU/mL nearly the same as the starting inoculum (*p* = 0.03; Fig 2). *E*. *coli* grew 3 log-fold in infant formula after 4 hours, whereas in PDHM grew 2.5 log-fold and in UDHM only grew 2 log-fold (*p* = 0.03; Fig 2). There was also a significant difference in *E*. *coli* growth in the PDHM and UDHM, where UDHM showed less growth of *E*. *coli* (*p* = 0.03; Fig 2).

### Bacterial growth in human breast milk supplemented with Lf

**Fig 3:**
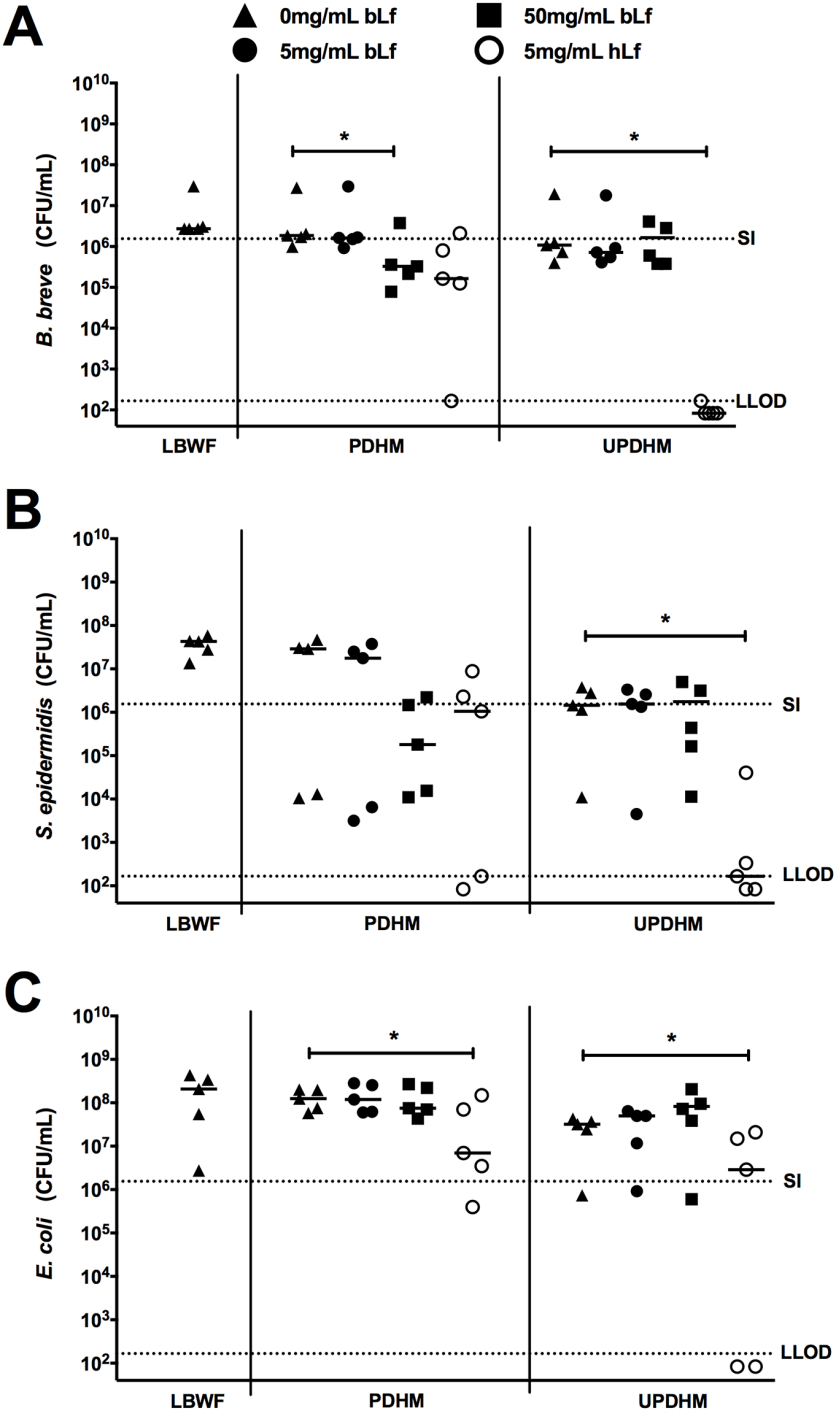
Growth of bacteria in donated human breast milk (DHM) spiked with bovine lactoferrin (bLf) and human lactoferrin (hLf). Results from five experiments showing median of the remaining CFU/mL of *B*. *breve* (A), *S*. *epidermidis* (B) and *E*. *coli* (C) after 4 hours of incubation in each condition. The starting inoculum was quantified at the start of each experiment and the median starting inoculum (SI) and lower limit of detection (LLOD) over the five experiments are indicated by the dotted lines. *p <0.05, comparing remaining bacterial CFU/mL in un/pasteurised DHM vs un/pasteurised DHM + bLf/hLf by Wilcoxon matched-pairs signed rank test.

We next examined bacterial growth in PDHM and UDHM supplemented with clinically relevant doses of bLf or hLf. *B*. *breve* growth remained unaffected by the presence of 5mg/mL of bLf in both PDHM and UDHM. However, at a bLf dose ten times higher (at 50mg/mL), growth of *B*. *breve* was significantly inhibited in PDHM (1 log-fold inhibition, *p* = 0.03; Fig 3A). In contrast, supplementation with hLf was more effective at inhibiting *B*. *breve* growth compared to bLf. In PDHM, 5mg/mL hLf caused more than twice the inhibition of *B*. *breve* growth when compared to the same concentration of bLf. This effect was even more pronounced in UDHM, where addition of hLf resulted in *B*. *breve* counts 4 log-fold lower compared to untreated UDHM (*p* = 0.03; Fig 3A,). In UDHM spiked with hLf, the remaining density of *B*. *breve* was lower than the lower limit of detection in each of the milk samples (>1x10^3^ CFU/mL).

A similar pattern of growth was seen for *S*. *epidermidis*. In PDHM, *S*. *epidermidis* growth remained unaffected when supplemented with bLf. However, *S*. *epidermidis* growth was significantly inhibited in UDHM when supplemented with hLf when compared with UDHM only (*p* = 0.03; Fig 3B). Bovine Lf did not inhibit growth of *E*. *coli* either in PDHM and UDHM. In contrast, growth of *E*. *coli* was significantly reduced by hLf, particularly in UDHM (*p* = 0.03; Fig 3C). Lastly, we co-cultured *B*. *breve* with either *S*. *epidermidis* or *E*. *coli*, under the same conditions as above and did not find significant differences in the growth of the bacteria in single or co-culture (see S1–S4 Figs).

## Discussion

This is the first study evaluating the effects of combining Lf and *B*. *breve* supplements in milk. Probiotic supplementation with *B*. *breve* may improve intestinal health and reduce LOS and NEC and the effects of prophylactic Lf administration are currently being investigated in large international studies. The design of this study was modelled around clinical scenarios within the NICU where these agents are commonly administered to infants in enteric feeds, including incorporation with breast milk. We found that 1) bLf has a negative impact of probiotic growth at higher doses, 2) UDHM has significantly greater antimicrobial activity than PDHM and 3) hLf has significantly greater antimicrobial activity than bLf.

Both hLf and high dose bLf were found to significantly inhibit growth of *B*. *breve*. The doses of hLf used in this study reflect those found for hLf in fresh human breast milk from mothers of preterm neonates as reported by Trend *et al* [9] and the doses of bLf reflect therapeutic doses used in current bLf trials. Many neonatal units globally administer routine probiotic supplements to preterm neonates with evidence of improved outcomes, including mortality, NEC and sepsis [23–27]. Previous studies have shown inconsistent growth modulation of *B*. *breve* in the presence of hLf and bLf, with some groups showing enhanced growth and others showing inhibition of growth [28–30]. The varying effects of Lf on *B*. *breve* could be attributed to inconsistencies in the differing concentrations and forms of Lf (i.e. iron-saturated vs iron-poor) used between studies. Additionally, these previous studies have assessed the effect of Lf on *B*. *breve* in different bacterial culture media, which may affect the sensitivity to Lf. In contrast, we have assessed the effect hLf and bLf on *B*. *breve* in human breast milk and have shown that they both have an inhibitory effect on *B*. *breve* survival and growth under these conditions. This may indicate that colonisation of the neonatal intestine by *Bifidobacterium* spp. could be reduced if milk is concurrently supplemented with bLf, the current form of Lf supplement under trial in preterm infants.

Unpasteurised human breast milk has been consistently found to have greater levels of antimicrobial activity and higher levels of Lf [8, 9, 31]. Our finding of lower Lf and less antimicrobial activity in PDHM are in accordance to other groups’ findings which examined the effects of unpasteurised and pasteurised human breast milk on the growth of pathogenic bacteria, including *E*. *coli*, *Pseudomonas aeruginosa* and *S*. *aureus*. [32–34]. Preterm infants preferentially receive unpasteurised mother’s own milk when in the NICU, and this has been consistently demonstrated to have a positive effect of developmental and immunological outcomes [31]. If mother’s own milk is not available or in inadequate supply, preterm infants receive donor breast milk which is pasteurised to eliminate possible transmission of infection [21]. Our findings, in combination with past studies, demonstrate that UDHM is far more effective at controlling sepsis pathogens and delivering important immune proteins, such as Lf, to preterm neonates [8, 9, 31]. Although UDHM was more effective at inhibiting *B*. *breve* growth compared with infant formula, the median remaining CFU/mL was approximately equal to the starting inoculum, and therefore *B*. *breve* may persist and be able to colonise the lower gut of the preterm infant. In addition to this, we found that the presence of viable *B*. *breve* did not affect the activity of supplemented Lf against the pathogenic bacteria tested (data not shown). Clinical reports stating that *B*. *breve* supplementation is safe and that viable counts persist in the stools of preterm infants [23, 35] are supported by our findings. Routine supplementation with *B*. *breve* is also associated with decreased NEC≥ Stage II and ‘NEC≥ Stage II or all-cause mortality’ in neonates <34 weeks [17]. Despite the concerns about the interaction between *B*. *breve* and Lf, the clinical data on use of this strain for routine probiotic supplementation in preterm infants are reassuring.

Human Lf combined with UDHM was far more effective at inhibiting *S*. *epidermidis*, *E*. *coli* and *B*. *breve* compared to the equivalent dose of bLf. The loss of the antimicrobial activity observed in our study with pasteurisation of human breast milk may also explain why bLf had less antimicrobial activity compared to the same dose of hLf. The thermal stability of the native form of bLf after heat treatment has been shown to denature at 62°C [36, 37], which is within the range used in the Holder pasteurisation method commonly used for most human breast milk sterilisation [21]. The bLf supplements we used were pasteurised, and this is something to consider before using during clinical trials. Future forms of human breast milk sterilisation that avoid heating (e.g. UV-C irradiation) may better preserve Lf stability and increase the antimicrobial activity of bLf supplements.

A study by Kim *et al*, showed that bLf supplemented culture media promoted *B*. *breve* growth where hLf had no effect on *B*. *breve* growth, however they did not evaluate the nature of this difference [29]. Our study used a dose of bLf and hLf fifty times greater than that of Kim *et al*, which explains why we saw consistent growth inhibition. The ability of hLf to significantly inhibit *S*. *epidermidis* and *E*. *coli* growth in UDHM is in accordance with findings by Trend *et al*, which also examined the activity of hLf in UDHM against these pathogens [9]. However this study did not draw comparisons between bLf and hLf. Griffiths *et al* showed that *E*. *coli* was significantly inhibited by 1.6mg/mL of both bLf and hLf, and that the iron-saturated form of bLf had no significant antimicrobial activity against *E*. *coli* [38]. We found that the growth *E*. *coli* was only significantly inhibited by hLf, which could explain the disparity in activity, if the bLf form we used was in fact iron saturated. Our hLf supplement was provided as 0.032% iron saturated content, whereas there were no iron saturation data provided about our bLf supplement.

Iron saturation content is an important factor for determining the antimicrobial activity and thermal stability of Lf [36, 37]. The more iron contained in a Lf supplement, the more thermal stability the Lf protein structure has [36, 37]. Conversely, the iron saturated, holo-form also has less antimicrobial activity than the iron poor apo-form [39]. This would suggest that there may be an optimal iron level for Lf that provides maximal stability and antimicrobial activity. Future studies should consider assessing the iron content of Lf supplements, potentially using mass spectrometry [40], and possibly assessing the available iron levels in the human breast milk samples to be supplemented in the NICU.

Our study is limited by the small sample size of available human breast milk specimens and limited number of representative probiotic and pathogenic bacterial strains tested. Future studies should examine the co-administration of Lf and a range of probiotic strains in a larger sample size of human breast milk samples treated under varied sterilisation conditions. Such *in vitro* studies may reveal optimal conditions for efficaciously combining these two supplements. There is also a need to further evaluate the fate of co-administered supplements *in vivo* over a range of times after administration. For example, examination of gastric aspirates and faecal bacterial loads after supplementation with Lf and probiotics could reveal the stability and interactions of both supplements before and after they enter the preterm gut. Such studies may help define the optimal window for delivery and repeated supplementation with maximal benefits for LOS and NEC prevention. In the meantime, our data suggest that it would be prudent, if supplementing with both probiotics and bLf, to schedule administration at separate times during feeding to avoid probiotic growth inhibition.

## Supporting information

S1 Fig Growth of *S*.*epidermidis* co-cultured with *B*. *breve* in donated human breast milk (DHM) spiked with bovine (bLf) and human lactoferrin (hLf). Results from five experiments showing median of the remaining CFU/mL of *S*. *epidermidis* when co-cultured with *B*. *breve* in pasteurised (A) and unpasteurised (B) donated human breast milk after 4 hours of incubation. The solid symbols indicate single culture of *S*. *epidermidis* and the open symbols indicate co-culture with *B*. *breve*. The starting inoculum was quantified at the start of each experiment and the median starting inoculum (SI) and the lower limit of detection (LLOD) over the five experiments are indicated by the dotted lines. *p <0.05, comparing bacterial CFU/mL in un/pasteurised DHM +/- bLf/hLf vs un/pasteurised DHM +/- bLf/hLf.+ *B*. *breve* by Wilcoxon matched-pairs signed ranked test. NB# For experiments using single bacterial species, the media used for enumeration was as previously listed above. However, for co-culture experiments (see supplementary findings) specialised media was made with reinforced clostridial agar, 0.05% L-cysteine HCl with the pH adjusted to 5.35 and incubated anaerobically in order to enumerate *B*. *breve* in the presence of *S*. *epidermidis* and *E*. *coli*.
(TIF)

S2 Fig Growth of *B*. *breve* co-cultured with *S*. *epidermidis* in donated human breast milk (DHM) spiked with bovine (bLf) and human lactoferrin (hLf). Results from five experiments showing median of the remaining CFU/mL of *B*. *breve* when co-cultured with *S*. *epidermidis* in pasteurised (A) and unpasteurised (B) donated human breast milk after 4 hours of incubation. The solid symbols indicate single culture of *B*. *breve* and the open symbols indicate co-culture with *S*. *epidermidis*. The starting inoculum was quantified at the start of each experiment and the median starting inoculum (SI) and the lower limit of detection (LLOD) over the five experiments are indicated by the dotted lines. *p <0.05, comparing bacterial CFU/mL in un/pasteurised DHM +/- bLf/hLf vs un/pasteurised DHM +/- bLf/hLf.+ *S*. *epidermidis* by Wilcoxon matched-pairs signed ranked test.
(TIF)

S3 Fig Growth of *E*. *coli* co-cultured with *B*. *breve* in donated human breast milk (DHM) spiked with bovine (bLf) and human lactoferrin (hLf). Results from five experiments showing median of the remaining CFU/mL of *E*. *coli* when co-cultured with *B*. *breve* in pasteurised (A) and unpasteurised (B) donated human breast milk after 4 hours of incubation. The solid symbols indicate single culture of *E*. *coli* and the open symbols indicate co-culture with *B*. *breve*. The starting inoculum was quantified at the start of each experiment and the median starting inoculum (SI) and the lower limit of detection (LLOD) over the five experiments are indicated by the dotted lines. *p <0.05, comparing bacterial CFU/mL in un/pasteurised DHM +/- bLf/hLf vs un/pasteurised DHM +/- bLf/hLf.+ *B*. *breve* by Wilcoxon matched-pairs signed ranked test.
(TIF)

S4 Fig Growth of *B*. *breve* co-cultured with *E*. *coli* in donated human breast milk (DHM) spiked with bovine (bLf) and human lactoferrin (hLf). Results from five experiments showing median of the remaining CFU/mL of *B*. *breve* when co-cultured with *E*. *coli* in pasteurised (A) and unpasteurised (B) donated human breast milk after 4 hours of incubation. The solid symbols indicate single culture of *B*. *breve* and the open symbols indicate co-culture with *E*. *coli*. The starting inoculum was quantified at the start of each experiment and the median starting inoculum (SI) and the lower limit of detection (LLOD) over the five experiments are indicated by the dotted lines. *p <0.05, comparing bacterial CFU/mL in un/pasteurised DHM +/- bLf/hLf vs un/pasteurised DHM +/- bLf/hLf.+ *E*. *coli* by Wilcoxon matched-pairs signed ranked test.
(TIF)
