# Supplementary figures and images for: Effects of lactoferrin on neonatal pathogens and Bifidobacterium breve in human breast milk
Source: PLoS One. 2018 Aug 22;13(8):e0201819. doi: 10.1371/journal.pone.0201819 (PMC6104981; doi:10.1371/journal.pone.0201819)

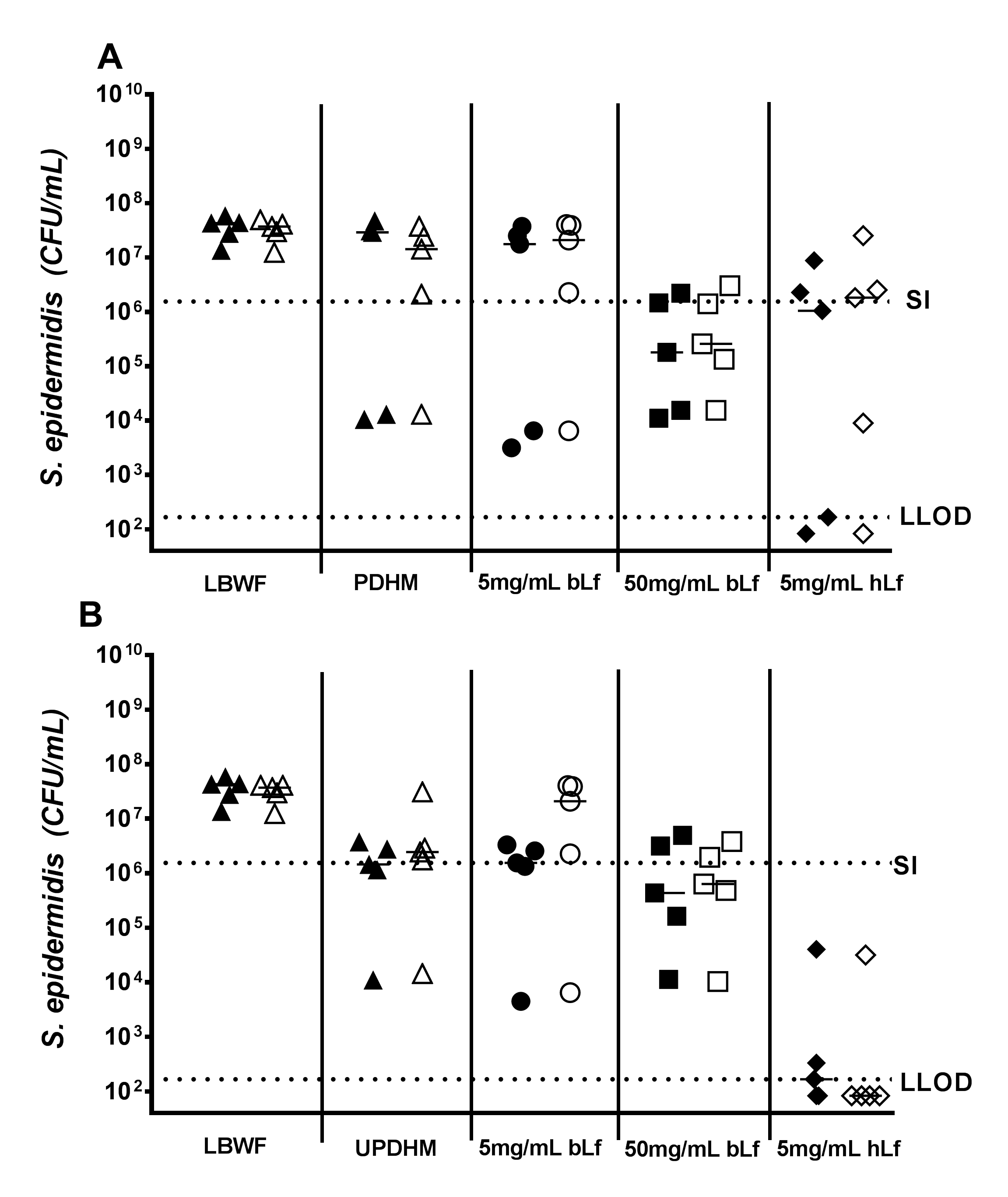

Supplement: S1 Fig — Results from five experiments showing median of the remaining CFU/mL of S. epidermidis when co-cultured with B. breve in pasteurised (A) and unpasteurised (B) donated human breast milk after 4 hours of incubation. The solid symbols indicate single culture of S. epidermidis and the open symbols indicate co-culture with B. breve. The starting inoculum was quantified at the start of each experiment and the median starting inoculum (SI) and the lower limit of detection (LLOD) over the five experiments are indicated by the dotted lines. *p <0.05, comparing bacterial CFU/mL in un/pasteurised DHM +/- bLf/hLf vs un/pasteurised DHM +/- bLf/hLf.+ B. breve by Wilcoxon matched-pairs signed ranked test. NB# For experiments using single bacterial species, the media used for enumeration was as previously listed above. However, for co-culture experiments (see supplementary findings) specialised media was made with reinforced clostridial agar, 0.05% L-cysteine HCl with the pH adjusted to 5.35 and incubated anaerobically in order to enumerate B. breve in the presence of S. epidermidis and E. coli. (TIF) [file pone.0201819.s001.tif]

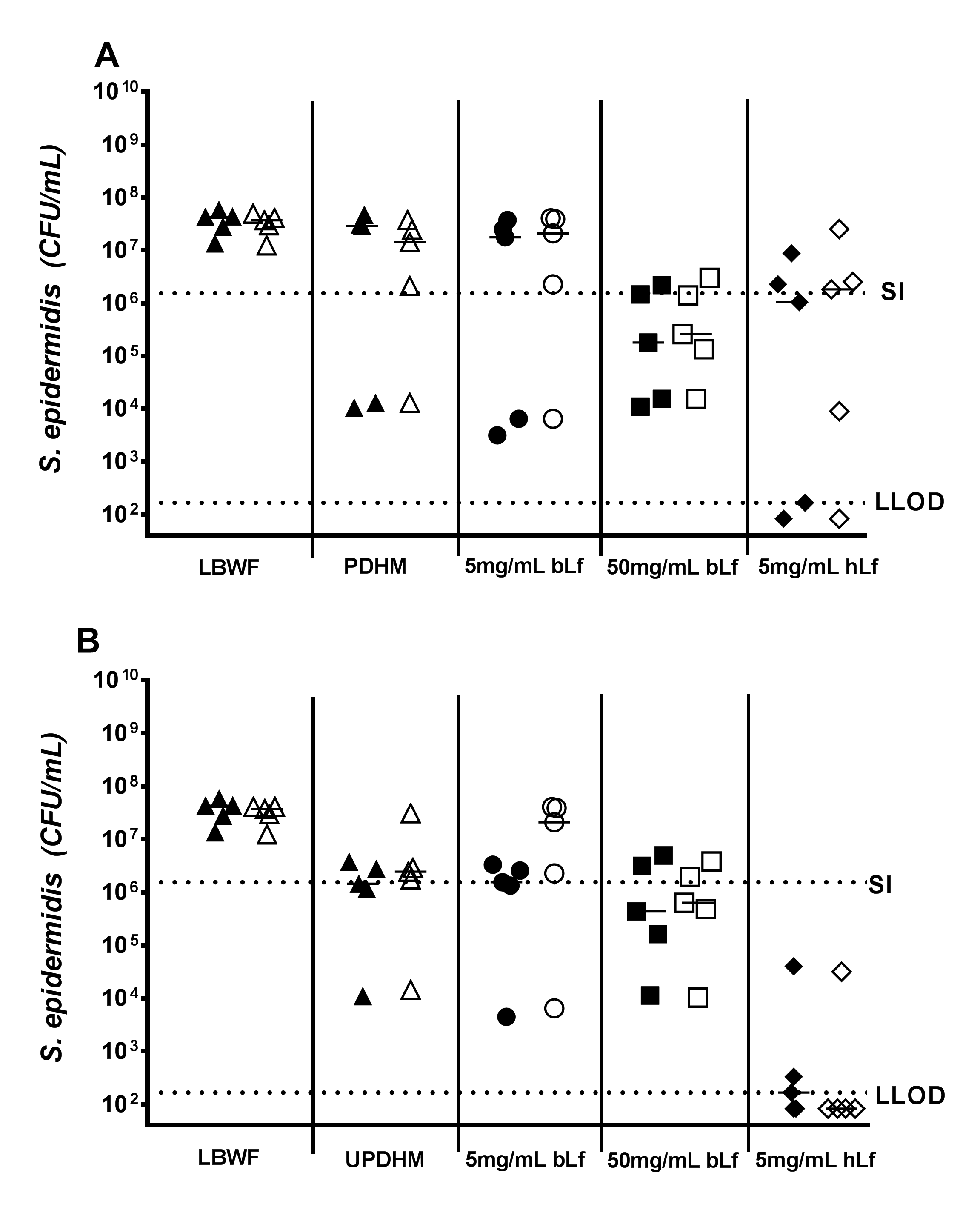

Supplement: S2 Fig — Results from five experiments showing median of the remaining CFU/mL of B. breve when co-cultured with S. epidermidis in pasteurised (A) and unpasteurised (B) donated human breast milk after 4 hours of incubation. The solid symbols indicate single culture of B. breve and the open symbols indicate co-culture with S. epidermidis. The starting inoculum was quantified at the start of each experiment and the median starting inoculum (SI) and the lower limit of detection (LLOD) over the five experiments are indicated by the dotted lines. *p <0.05, comparing bacterial CFU/mL in un/pasteurised DHM +/- bLf/hLf vs un/pasteurised DHM +/- bLf/hLf.+ S. epidermidis by Wilcoxon matched-pairs signed ranked test. (TIF) [file pone.0201819.s002.tif]

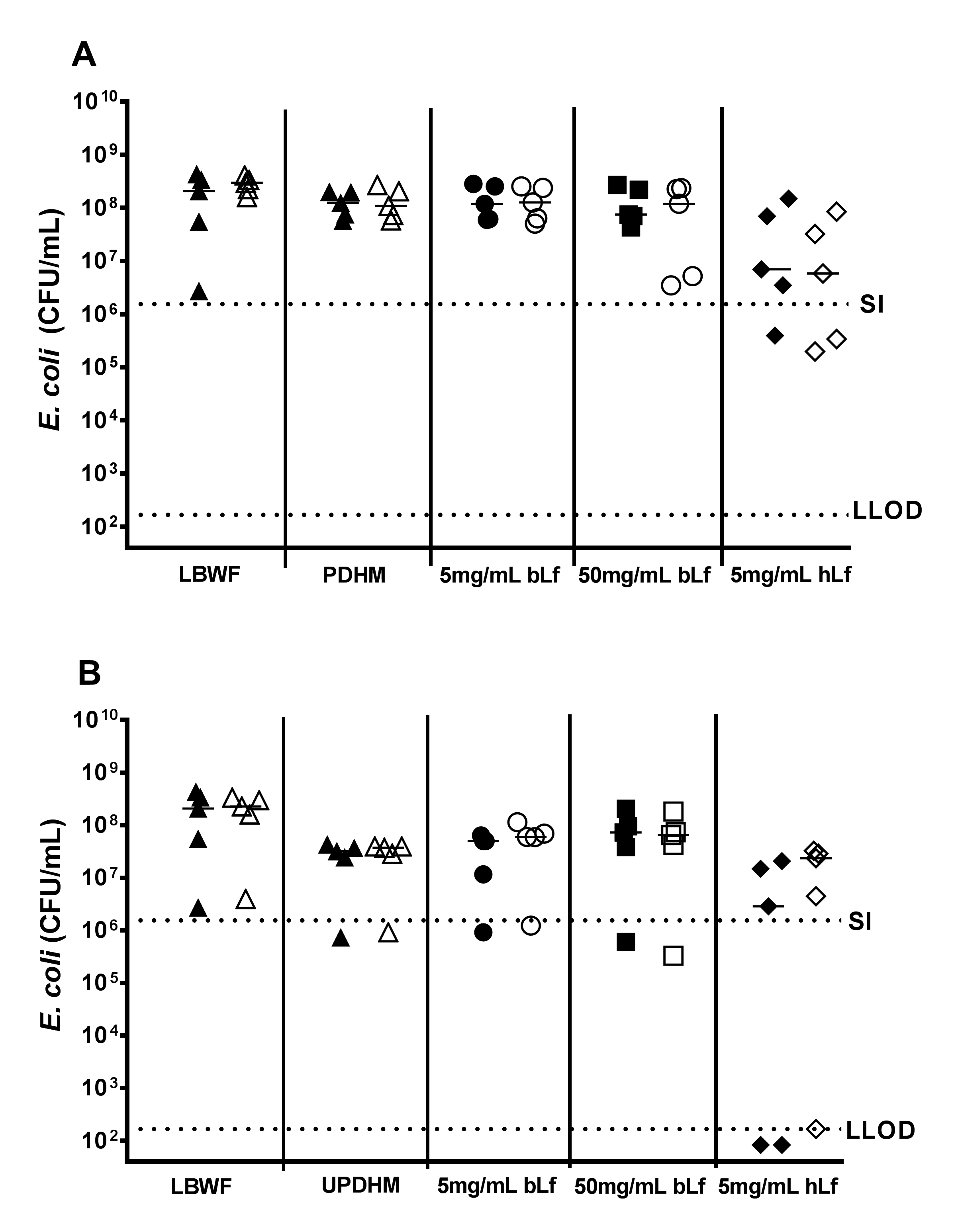

Supplement: S3 Fig — Results from five experiments showing median of the remaining CFU/mL of E. coli when co-cultured with B. breve in pasteurised (A) and unpasteurised (B) donated human breast milk after 4 hours of incubation. The solid symbols indicate single culture of E. coli and the open symbols indicate co-culture with B. breve. The starting inoculum was quantified at the start of each experiment and the median starting inoculum (SI) and the lower limit of detection (LLOD) over the five experiments are indicated by the dotted lines. *p <0.05, comparing bacterial CFU/mL in un/pasteurised DHM +/- bLf/hLf vs un/pasteurised DHM +/- bLf/hLf.+ B. breve by Wilcoxon matched-pairs signed ranked test. (TIF) [file pone.0201819.s003.tif]

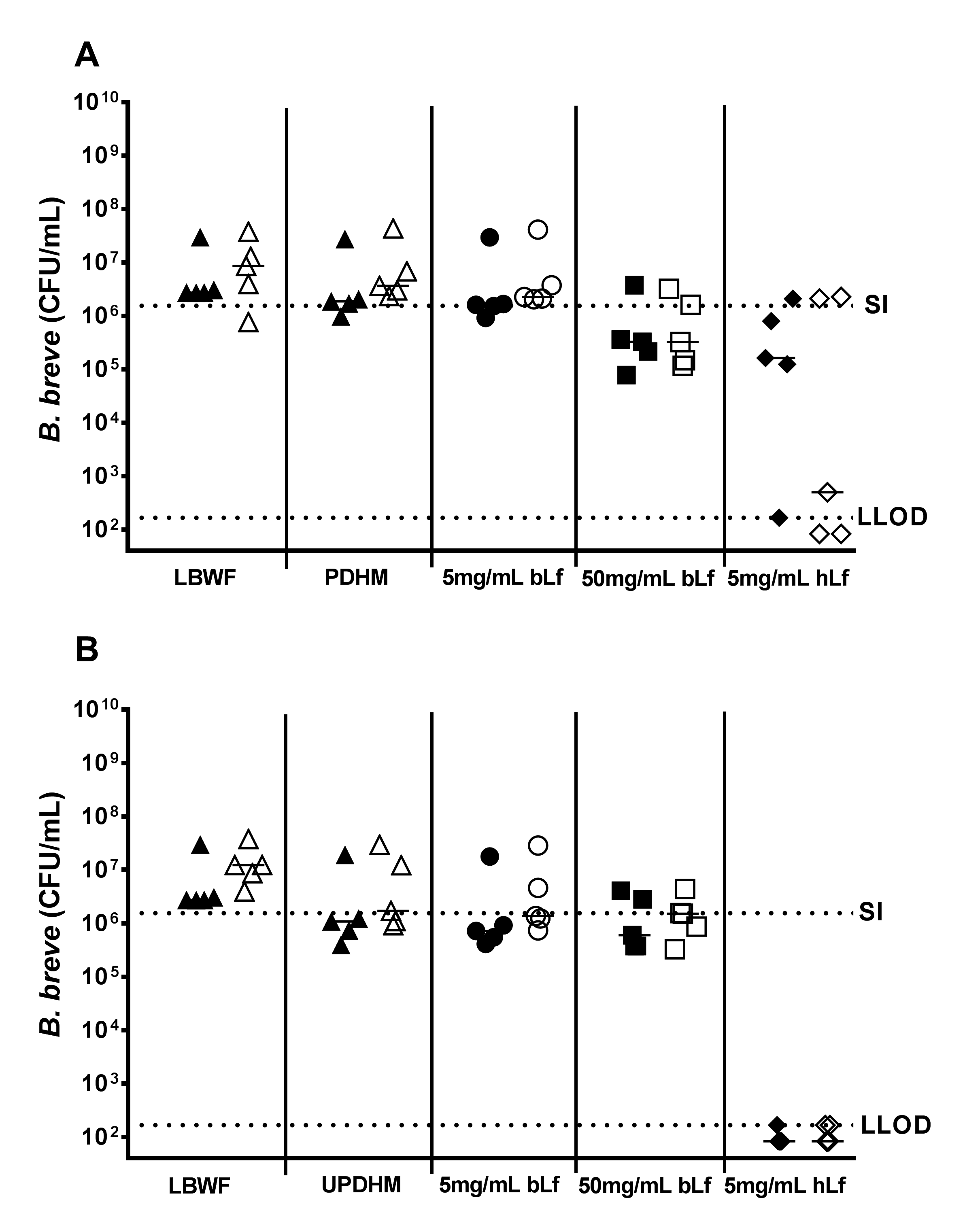

Supplement: S4 Fig — Results from five experiments showing median of the remaining CFU/mL of B. breve when co-cultured with E. coli in pasteurised (A) and unpasteurised (B) donated human breast milk after 4 hours of incubation. The solid symbols indicate single culture of B. breve and the open symbols indicate co-culture with E. coli. The starting inoculum was quantified at the start of each experiment and the median starting inoculum (SI) and the lower limit of detection (LLOD) over the five experiments are indicated by the dotted lines. *p <0.05, comparing bacterial CFU/mL in un/pasteurised DHM +/- bLf/hLf vs un/pasteurised DHM +/- bLf/hLf.+ E. coli by Wilcoxon matched-pairs signed ranked test. (TIF) [file pone.0201819.s004.tif]
